# Darbepoetin Alpha Reduces Oxidative Stress and Chronic Inflammation in Atherosclerotic Lesions of Apo E Deficient Mice in Experimental Renal Failure

**DOI:** 10.1371/journal.pone.0088601

**Published:** 2014-02-28

**Authors:** Nicole Arend, Karl F. Hilgers, Valentina Campean, Britta Karpe, Nada Cordasic, Bernd Klanke, Kerstin Amann

**Affiliations:** 1 Department of Internal Medicine- Nephrology, University of Erlangen-Nürnberg, Erlangen, Germany; 2 Department of Nephropathology, University of Erlangen-Nürnberg, Erlangen, Germany; Max-Delbrück Center for Molecular Medicine (MDC), Germany

## Abstract

**Background:**

Cardiovascular morbidity and mortality is very important in patients with chronic renal failure. This occurs even in mild impairment of renal function and may be related to oxidative stress and chronic inflammation. The nephrectomized apo E knockout mouse is an accepted model for evaluating atherosclerosis in renal dysfunction. Erythropoietin derivates showed anti-oxidative and anti-inflammatory effects. Therefore, this study evaluates the effects of Darbepoetin on markers of oxidative stress and chronic inflammation in atherosclerotic lesions in apo E knockout mice with renal dysfunction.

**Methods:**

Apo E knockout mice underwent unilateral (Unx, n = 20) or subtotal (Snx, n = 26) nephrectomy or sham operation (Sham, n = 16). Mice of each group were either treated with Darbepoetin or saline solution, a part of Snx mice received a tenfold higher dose of Darbepoetin. The aortic plaques were measured and morphologically characterized. Additional immunhistochemical analyses were performed on tissue samples taken from the heart and the aorta.

**Results:**

Both Unx and Snx mice showed increased expression of markers of oxidative stress and chronic inflammation. While aortic plaque size was not different, Snx mice showed advanced plaque stages when compared to Unx mice. Darbepoetin treatment elevated hematocrit and lowered Nitrotyrosin as one marker of oxidative stress, inflammation in heart and aorta, plaque stage and in the high dose even plaque cholesterol content. In contrast, there was no influence of Darbepoetin on aortic plaque size; high dose Darbepoetin treatment resulted in elevated renal serum parameters.

**Conclusion:**

Darbepoetin showed some protective cardiovascular effects irrespective of renal function, i.e. it improved plaque structure and reduced some signs of oxidative stress and chronic inflammation without affecting plaque size. Nevertheless, the dose dependent adverse effects must be considered as high Darbepoetin treatment elevated serum urea. Elevation of hematocrit might be a favorable effect in anemic Snx animals but a thrombogenic risk in Sham animals.

## Introduction

The prevalence and incidence of chronic kidney diseases (CKD) have been continually increasing worldwide [1]. Nowadays, cardiovascular events are the most important cause of morbidity and mortality in patients with CKD [2]. The incidence of myocardial infarction is threefold increased in CKD patients [3] and sevenfold increased in patient on hemodialysis [4] when compared to a matched healthy population. Several studies showed that the elevation of risk starts even in mild impairment of renal function [5], [6].

CKD is regarded as a pro-inflammatory state and associated with increased levels of oxidative stress [7], [8]. Compared with a renal healthy population CKD patients show elevated local markers of oxidative stress like Nitrotyrosine (NT) [9] and systemic inflammatory markers [10] like C-reactive protein (CRP) [11]. Pro-inflammatory cytokines and oxidative stress lead to endothelial dysfunction [8] and to formation of fatty streaks, the early stage of atherosclerosis [12]. Subsequent cell adhesion molecules like intercellular adhesion molecule (ICAM) and vascular cell adhesion molecule (VCAM) promote the formation of foam cells and atherosclerotic plaques [13]. Inflammatory molecules influence each other in a complex cascade while forming an atheroma [14]. In the late phase interaction of CD40 and CD154 [15] may lead to more advanced, more calcified plaques, activation of matrix metalloproteases and destabilization of the plaques [10] may occur with the risk of rupture and thrombembolia [16]. This risk is much higher in CKD than in renal healthy patients [17]. Therefore patients with CKD should be considered a high risk group for cardiovascular diseases [18].

As rodents do not develop atherosclerosis spontaneously, even when nephrectomized, Buzello et al. [19] introduced the models of uni- and subtotally nephrectomized apo E knockout mice for studying atherosclerosis in CKD showing a more aggressive morphology of atherosclerotic plaques which increased in relation to renal function impairment [19]. Bro et al. [20] and others [21] confirmed these findings and proofed the involvement of inflammation and oxidative stress via studying the change of cell adhesion molecules [22], NT [23] or CRP [24]. Therefore, apo E knockout mice are an accepted model for studying atherosclerosis in CKD., Of note, atherosclerotic lesions in these mice are very similar to those in human beings [25], although the presentation of cardiovascular disease in patients with renal disease is often atypical [26]. The apo E defect also leads to a dysregulation of the anti-oxidative [27] and anti-inflammatory system [28] and therefore to an advanced in-situ deposition of NT [29], a marker of oxidative stress, and increased aortic expression of the adhesions molecules ICAM and VCAM [20] in these mice.

Erythropoietin (Epo) derivates are widely known as therapeutic agents in the treatment of anemia, especially of the renal form [30]. It was recognized that cardiac function and capacity of patients treated with Epo was increased [31]. Further studies were undertaken to examine other possible protective effects of Epo derivates, i.e. anti-apoptotic effects via inhibiting caspase 3 [31] and stabilizing the BAX/Bcl-2-ratio in apo E deficient mice [32], even in chronic renal failure [33]. Furthermore Epo diminishes the production of inflammatory cytokines [34] and oxidative stress [35]. Finally, Epo showed significant beneficial effects on the morphology of atherosclerotic lesions of apo E knockout mice via reducing the lipid content of macrophages and therefore foam cell formation [36]. On the other hand it is important to consider possible side effects of Epo like rapid elevation of hematocrit [37], elevation of blood pressure [38] or an increased risk of thrombotic emboli [38] via activation of thrombocytes [39]. Therefore, Epo derivates were developed in order to profit from these protective effects without being at risk of thromboembolia. Darbepoetin is a long acting derivate of Epo which shows protective effects in doses where it doesn't elevate hematocrit [40]. Higher doses however do elevate hemoglobin, which can lead to disturbed blood flow and therefore increases risk of thrombembolic events, as shown in the TREAT study [41]. Low dose Darbepoetin alpha also showed protective effects on renal [42] and cardiac function [43]. Dursun et al. investigated the effect of Darbepoetin alpha on atherosclerosis in apo E knockout mice without CKD and showed changes in proteomic profile of treated mice although plaque morphology was not affected [44].

Therefore, it was the aim of the present study to evaluate possible anti-oxidative and anti-inflammatory effects of two different doses of Darbepoetin alpha on atherosclerotic lesions in Apo E knockout mice with and without renal function impairment.

## Materials and Methods

### Animals and study design

Sixty-two apo E knockout mice were held at constant temperature of 22°C and 50–60% humidity. The animals had free admittance to standard diet (5% fat, 48% carbohydrates, 22.5% proteins) and water. At the age of three months mice were randomly distributed to either unilateral (Unx), subtotal nephrectomy (Snx) or sham operation (Sham) by a person not knowing any laboratory findings of them.. Therefore, the left kidney was removed, weighed and one week later a part of the right kidney was also taken out corresponding to 2/3 of the weight of the left kidney for Snx. For Sham operation the kidney capsule was opened without withdrawal of any tissue. After another week each group was divided in the following subgroups: One received saline solution subcutaneous, one darbepoetin alpha at a dose of 11 µg/kg body weight (“normal dose”) once a week for four months. In the Snx-Group there was a third subgroup receiving 110 µg/kg body weight Darbepoetin alpha (“high dose”) once a week for four months. These doses were chosen according to the work of Egrie et al [45] where such doses caused at most a very modest elevation of hematocrit in mice. The exact number of animals per group and of animals that died during the study is shown in table S1. The experiment was terminated after four months of treatment, i.e. when animals were seven months of age, by perfusion fixation.

In contrast to Erythropoietin, which is traditionally measured in activity units (U), the doses of Darbepoetin alpha are expressed in µg. 1 µg of Darbepoetin alpha equals 200 U of Erythropoietin.

### Ethics

All procedures performed on animals were done in accordance with the NIH Guide for the Care and Use of Laboratory Animals and were approved by the local government authorities (Regierung von Mittelfranken, approval number AZ # 621-2531.31-13/03) after evaluation by the local government's review board for animal research ethics. All surgery was performed under isoflurane anesthesia, and all efforts were made to minimize suffering. If judged necessary by a veterinarian, buprenorphine hydrochloride was injected to prevent of relief suspected pain or discomfort.

### Measurement of body weight, blood pressure, hematocrit, serum and urinary parameters

Body weight was determined once per week, before blood pressure measurements and before perfusion fixation using a high precision scale. Blood pressure was measured via an intraarterial catheter under isofluran anesthesia. Hematocrit was determined every two weeks in whole blood from V. saphena magna, serum parameters were analyzed right before perfusion fixation via centrifugation of EDTA whole blood and urinary parameters were determined from urine collected 24 h before perfusion fixation. Creatinine clearance was calculated by measuring the concentration of creatinine in serum and urine over 24 hours.

### Tissue preparation, morphologic and morphometric investigations

The study was terminated by perfusion fixation. Briefly, the left ventricle was punctured and flushed with 10% rheomacrodex to dilate the vessels and prevent interstitial edema. Then the vascular system was rinsed with 0.9% NaCl for 10 minutes followed by rinsing with glutaraldehyd-acid-buffer to harden tissue. After 5 min perfusion was stopped and the heart, the aorta and other organs were taken out, fixed in 4% buffered formalin and embedded in paraffine.

The aorta was sectioned into 3 µm thick paraffine sections, every tenth section was stained with hematoxylin-eosin (HE) and used for morphometric analyses. Thickness and area of aortic media was measured using a semiautomatic image analyzing system (analySIS®) by building the arithmetic mean of 10 measurements. Total plaque volume was determined by multiplying plaque area with the length of plaque containing aorta ascendens. In the section next to the aortic valve plaque stage and cholesterol crystals content were determined according to the classifications in tables 1 [46] and 2.

**Table 1:** Plaque stages according to Rosenfeld et al. [46].

Score	Criteria
1	Early plaque with foam cells
2	Plaque with cholesterol crystals
3	Advanced plaque with loss of lumen or fibrous cap, hemorrhage, chondrocyte-like cells, layering or necrosis

**Table 2:** Definition of the cholesterol-score.

Score	Criteria
0	No cholesterol crystals
1	Few cholesterol crystals (approx. 25% of plaque area)
2	Several cholesterol crystals (approx. 50% of plaque area)
3	Many cholesterol crystals (approx. 75% of plaque area)

### Immunhistochemistry

Proteine expression of Nitrotyrosine (NT), ICAM, VCAM, CRP, CD 40 and CD154 in aortic plaques and intramyocardial arteries was determined by semiquantitative scoring (score 0–4) of immunohistochemical staining (tables 3 and 4).

**Table 3:** Definition of plaque-score.

Score	Expression
0	No expression
1	Weak expression (up to 25% dyeing)
2	Middle expression (up to 50% dyeing)
3	Strong expression (up to 75% dyeing)
4	Very strong expression (more than 75% dyeing)

**Table 4:** Definition of myocardial artery expression-score.

Score	Expression
0	0 of 4 myocardial arteries were dyed
1	1 of 4 myocardial arteries were dyed
2	2 of 4 myocardial arteries were dyed
3	3 of 4 myocardial arteries were dyed
4	4 of 4 myocardial arteries were dyed

Aortic endothelium was evaluated as whether being stained (1) or not (0) and the percentage of positively stained endothelia was used for statistical comparisons.

Data regarding the used antibodies is provided in table S2.

### Statistics

All statistical analyses were made with SPSS 21, SPSS Inc. Data are given in mean ± standard deviation apart from the immunhistochemical analyses which are provided as scatterplots. The non-parametric Kruskal-Wallis-Test followed by Fisher's exact test posthoc was used for comparison of means. Correlations were calculated with Pearson's correlation coefficient. Differences were considered significant when p<0.05.

## Results

### Animal data

Baseline hematocrit of saline and Darbepoetin treated animals was significantly lower in Snx than in Sham or Unx animals, the latter two groups did not differ. Mean hematocrit after Darbepoetin alpha treatment was significantly higher than baseline hematocrit in all groups. The changes in hematocrit after saline treatment were not significant (table 5). Body weight did not differ between the groups. In the treatment group, and partly by trend in the control group, there was a significant increase in serum-urea and serum-creatinine from Sham to Unx to Snx, accompanied by a significant decrease in urine-creatinine concentration, resulting in a significantly worse creatinine-clearance, i.e. glomerular filtration rate (table 6). Darbepoetin treatment led to further significant reduction of urine creatinine concentration in Unx and Snx animals, especially some Snx animal treated with the high dose of Darbepoetin alpha showed very high levels of serum-urea, but to a significantly better glomerular filtration rate in the Sham group. On blood pressure, food or water consumption, albuminuria, albumin, phosphate, serum cholesterol or lipids neither nephrectomy nor Darbepoetin alpha treatment had any effect (table 7).

**Table 5:** Hematocrit.

	Baseline (%)	After Darbepoetin treatment (%)	After Saline treatment (%)
**Sham**	51.7±3.08	60.8±8.13	52.9±3.50
**Unx**	51.1±3.64	59.8±9.07	53.9±6.88
**Snx**	44.8±2.08	59.6±7.88	49.1±8.89
**Snx high**	37.6±2.97	64.1±8.74	-
**Kruskal-Wallis**	p<0.001	n.s.	n.s.
**Fisher Operation Sham/Unx/Snx**	p<0.001	n.s.	p<0.05
**Fisher before - after treatment**		p<0.05 (Sham/Unx)p<0.001 (Snx)	n.s.

*Mean ± standard deviation.*

n.s. = not significant;

**Table 6:** Urea and creatinine.

		Serum-urea (mg/dl)	Serum-creatinine (mg/dl)	Urine-creatinine (mg/dl)	Creatinine clearance (ml/min)
**Sham**	**Control**	51.4±15.0	0.58±0.70	29.6±15.5	0.13±0.17#
	**Darbepoetin**	40.4±15.9	0.08±0.02	28.5±6.58°	0.42±0.20
**Unx**	**Control**	83.4±42.7	0.26±0.22	28.9±8.12#	0.29±0.40
	**Darbepoetin**	69.3±22.1*	0.11±0.02	20.5±6.25°	0.30±0.07*
	**Control**	87.3±12.0	0.17±0.04	24.8±8.33#	0.16±0.09
**Snx**	**Darbepoetin**	79.9±10.4*	0.16±0.03*	13.2±4.47	0.17±0.08*∼
	**Darbepoetin high**	108±35.2	0.24±0.11	17.2±3.26	0.11±0.06
**Kruskal-Wallis**		p<0.001	p<0.01	p<0.01	p<0.05
**Fisher Operation Sham/Unx/Snx**		* p<0.001 vs. Sham DarbepoetinControl n.s.	* p<0.001 vs. Sham DarbepoetinControl n.s.	° p<0.001 vs. Snx DarbepoetinControl n.s.	* p<0.001 vs. Sham Darbepoetin∼ p<0.001 vs. Unx Darbepoetin
**Fisher Medication Control/Darbepoetin**		n.s.	n.s.	Sham n.s. #p = 0.05 vs. Darbepoetin respectively	# p<0.05 vs. Sham DarbepoetinUnx, Snx n.s.

*Mean ± standard deviation.*

n.s. = not significant.

**Table 7:** Triglycerides and cholesterol.

		Triglycerides (mg/dl)	Whole Cholesterol (mg/dl)	LDL-Cholesterol (mg/dl)
**Sham**	**Control**	91.8±39.2	277±91.9	181±69.5
	**Darbepoetin**	82.7±36.7	298±72.3	167±32.5
**Unx**	**Control**	83.8±45.7	321±105	188±60.6
	**Darbepoetin**	70.0±33.4	389±123	248±73.3
	**Control**	89.2±24.0	393±55.8	251±70.7
**Snx**	**Darbepoetin**	78.6±25.2	299±60.2	188±57.4
	**Darbepoetin high**	288±157	436±249	265±195
**Kruskal-Wallis**		n.s.	n.s.	n.s.
**Fisher Operation Sham/Unx/Snx**		n.s.	n.s.	n.s.
**Fisher Medication Control/Darbepoetin**		n.s.	n.s.	n.s.

*Mean ± standard deviation.*

**n.s. = not significant.**

### Plaque morphometry

**Figure 1:**
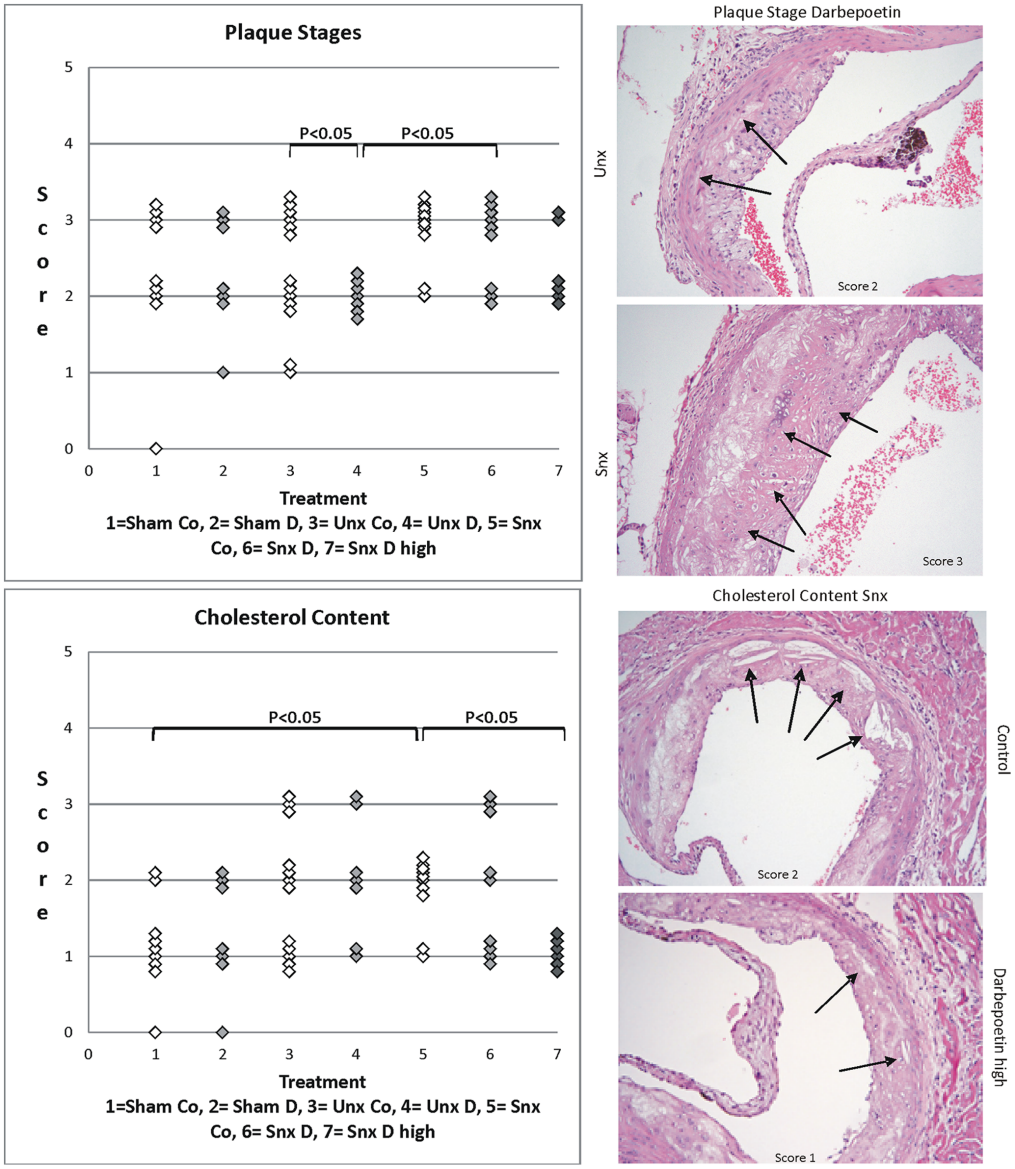
Plaque stages and cholesterol content. Left row shows scatter plots of scores of plaque stage and plaque cholesterol content of the seven treatment groups. Significant differences are marked with the certain significance level. Right row shows histological examples. Plaque stage of Darbepoetin alpha treated Unx animal equals score 2 (arrows show cholesterol crystals) while plaque stages of Snx animal equals score 3 (arrows show chondrocyte-like cells and necrosis). Cholesterol content of control Snx animal equals score 2 and that of Snx animal treated with high dose Darbepoetin alpha equals score 1. Arrows show cholesterol crystals.

Mean plaque area, plaque volume, media thickness and media area showed no significant differences between the groups (table 8). Although plaque morphology was not significantly different Snx animals showed significantly more advanced plaque stages than Unx and also significantly more cholesterol crystals in the plaque than Sham animals. Most importantly, Darbepoetin alpha treatment significantly lowered plaque stage in the Unx group and the amount of intraplaque cholesterol crystals in the Snx high treatment group (fig. 1).

**Table 8:** *Morphometry*.

		Plaque area (*10^3^ µm^2^)	Plaque volume (*10^6^ µm^3^)	Media thickness (µm)	Media area (*10^3^ µm^2^)
**Sham**	**Control**	104±86.1	11.2±7.70	87.2±16.4	295±49.6
	**Darbepoetin**	104±67.7	15.0±11.6	88.3±13.5	301±33.4
**Unx**	**Control**	108±63.8	15.5±9.60	87.5±10.8	315±34.5
	**Darbepoetin**	112±70.3	16.5±10.7	93.6±9.62	336±53.6
	**Control**	79,1±7.73	16.3±8.60	90.7±7.90	302±71.0
**Snx**	**Darbepoetin**	170±84.4	24.9±13.2	84.1±7.62	268±58.0
	**Darbepoetin high**	101±35.4	15.2±5.30	94.4±6.27	335±54.8
**One-way AOVA**		n.s.	n.s.	n.s.	n.s.
**Two-way ANOVA Operation Sham/Unx/Snx**		n.s.	n.s.	n.s.	n.s.
**Two-way ANOVA Medication Control/Darbepoetin**		n.s.	n.s.	n.s.	n.s.

*Mean ± standard deviation.*

n.s. =  not significant;

### Nitrotyrosine (NT) as a marker of oxidative stress

**Figure 2:**
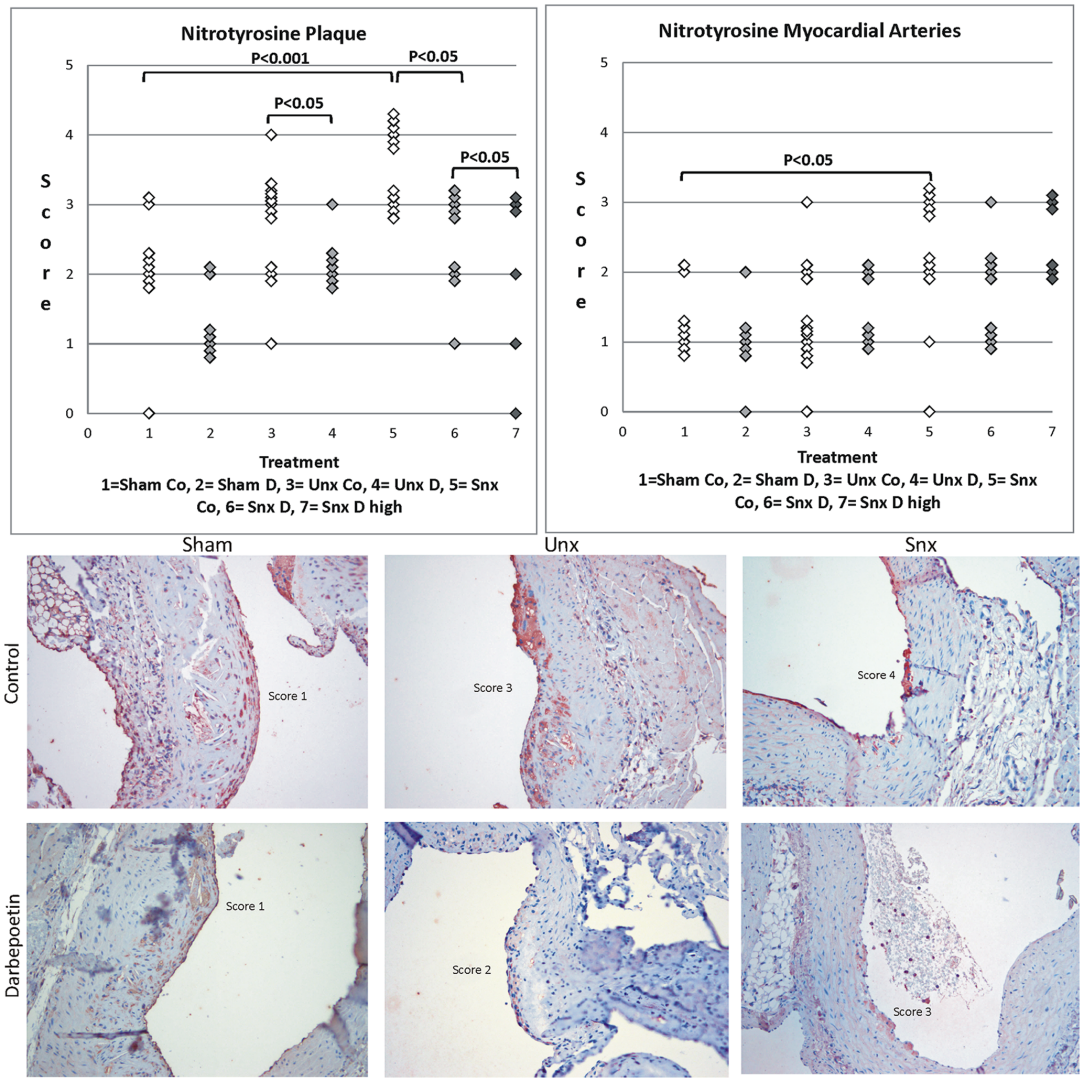
Nitrotyrosine plaque and myocardial artery score. Upper row shows nitrotyrosine staining score of plaque and intramyocardial arteries. Significant differences are marked with the certain significance level. Lower row shows histological examples. Plaque staining increases stepwise from Sham to Snx. Darbepoetin alpha treated examples show lower scores in each group.

**Figure 3:**
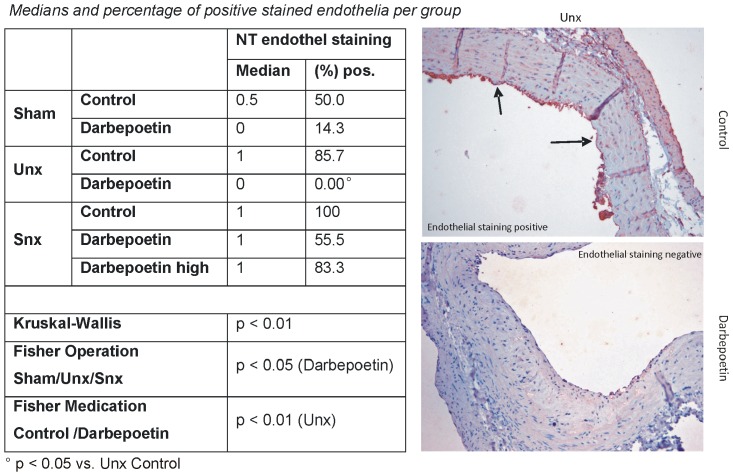
Nitrotyrosine aortic endothelium score. Table shows nitrotyrosine staining score of aortic endothelia (0 or 1) and percentage of positively stained endothelia per group. Significant differences are marked with the certain significance level. Right row shows histological examples. The score of the Unx control animal equals 1 (arrows show stained areas of the endothelium) while the score of the Darbepoetin alpha treated Unx animal equals 0.

The degree of NT staining in aortic plaques was significantly influenced by impairment of renal function. It increased stepwise from Sham to Unx to Snx with a significant difference between Sham and Snx animals. Also the intramyocardial arteries showed a significantly stronger expression of NT in parallel with lower renal function (fig. 2). In the Darbepoetin alpha group Snx animals showed a significantly higher percentage of NT positive aortic endothelium than Unx animals (fig. 3). Of note, NT staining in intramyocardial arteries correlated significantly positive with aortic media thickness (p<0.05) and serum urea concentration (p<0.01). Other markers of renal function showed correlations to NT expression in plaque (with creatinine clearance: p = 0.06) or intramyocardial arteries (with urine creatinine: p = 0.06; with creatinine clearance: p = 0.07) by trend. Darbepoetin alpha treatment also significantly influenced the protein expression of NT in aortic plaques. In Snx animals a normal dose of Darbepoetin alpha and additionally a high dose led to significantly lower in-situ NT staining (fig. 2). No influence of Darbepoetin, however, was seen on expression of NT in intramyocardial arteries. Darbepoetin alpha treatment significantly lowered the percentage of NT positive endothelium in Unx animals (fig. 3).

### Markers of chronic inflammation

#### ICAM (Intercellular Adhesion Molecule)

**Figure 4:**
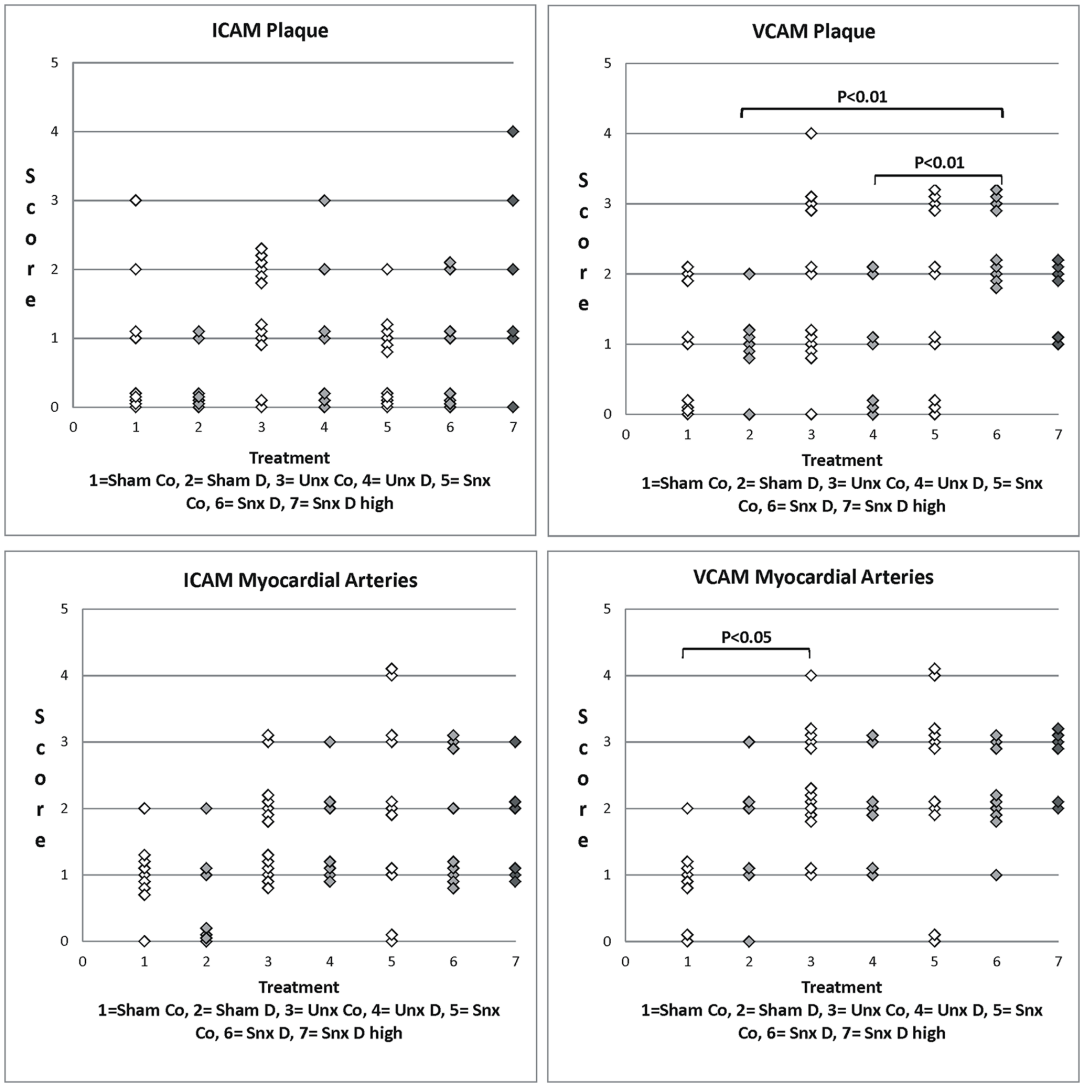
ICAM and VCAM plaque and myocardial artery score. Scatter plots of ICAM and VCAM staining score of plaque and intramyocardial arteries of the seven treatment groups. Significant differences are marked with the certain significance level.

The protein expression of ICAM in the aorta did not differ significantly between the nephrectomy or treatment groups, neither in the intramyocardial arteries or the aortic plaque (fig. 4) nor in the aortic endothelium (data not shown).

#### VCAM (Vascular Cell Adhesion Molecule)

VCAM in intramyocardial arteries and aortic plaques was significantly higher in Unx and Snx animals than in Sham (fig. 4). Of note, expression in Snx was also significantly higher than in Unx. Expression in the aortic plaques correlated negatively with the urinary protein concentration. In the aortic endothelium there was no significant difference (data not shown). Interestingly, Darbepoetin alpha treatment did not affect VCAM expression in the heart or the aorta.

#### CRP (C-reactive protein)

**Figure 5:**
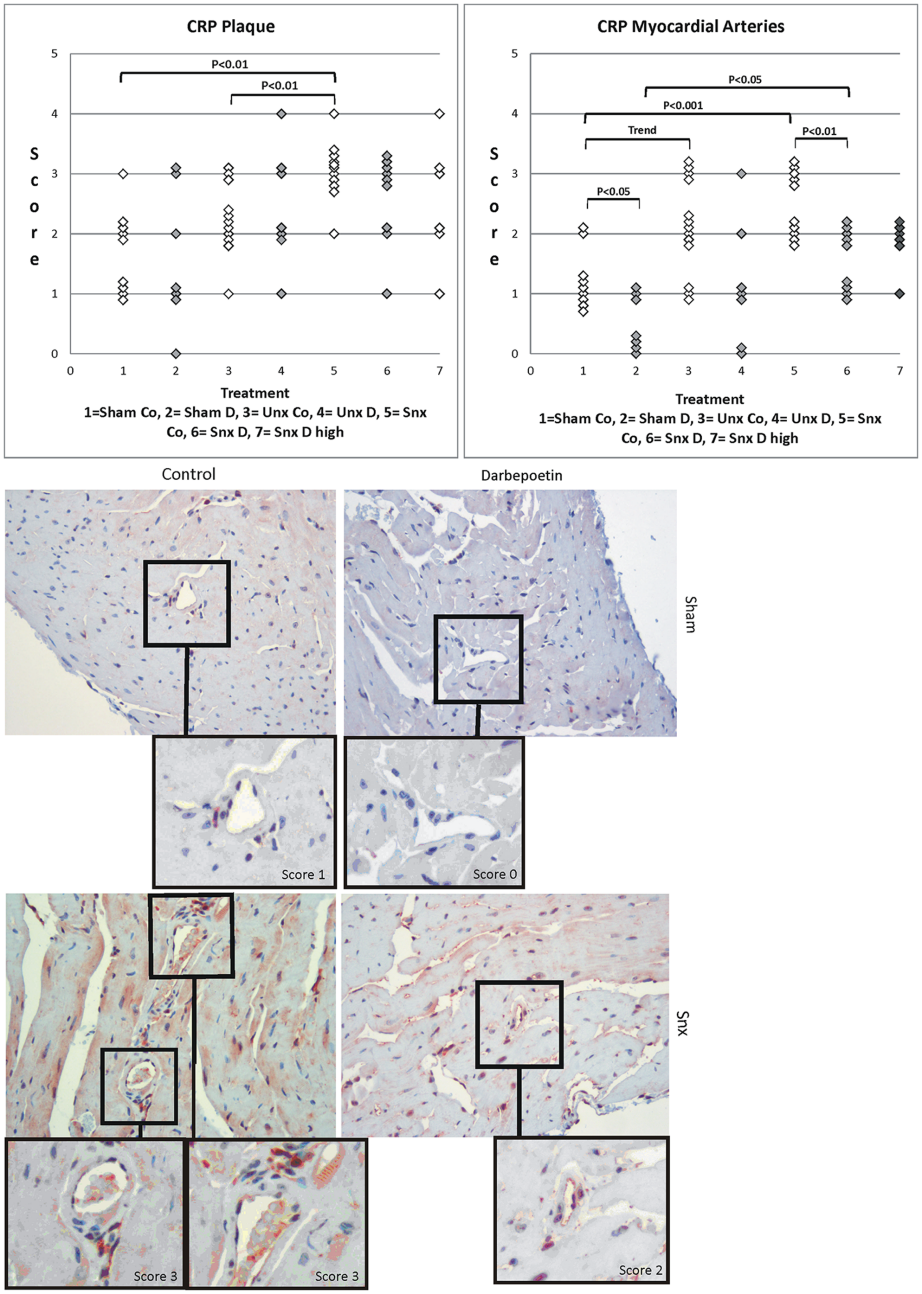
CRP plaque and myocardial artery score. Upper row shows CRP staining score of plaque and intramyocardial arteries. Significant differences are marked with the certain significance level. Lower row shows histological examples. Myocardial artery staining increases from Sham to Snx. Darbepoetin alpha treated examples show lower scores in each group.

Nephrectomy showed a significant influence on in-situ CRP protein expression in plaques and intramyocardial arteries. There was a significantly higher CRP expression in plaques in Unx and Snx mice than in Sham and a stepwise increased expression from Sham over Unx to Snx in intramyocardial arteries. CRP expression in intramyocardial arteries correlated significantly with serum urea concentration (p<0.01). Here, Darbepoetin alpha treatment significantly lowered CRP expression in Sham and Snx animals (fig. 5). In the aortic plaques no such effect was seen.

In the aortic endothelium no significant effect of nephrectomies or Darbepoetin alpha treatment was seen (table 9).

**Table 9:** Expression of CRP, CD40 and CD154 in aortic endothelium.

		CRP staining		CD 40 staining		CD154 staining	
		Median	(%) pos.	Median	(%) pos.	Median	(%) pos.
**Sham**	**Control**	0	20.0	0	20.0	0	20.0
	**Darbepoetin**	0	14.3	0	28.6	0	42.9
**Unx**	**Control**	0	42.9	0	42.9	0	42.9
	**Darbepoetin**	0	28.6	1	85.7	0	28.6
	**Control**	1	100	1	100	1	66.6
**Snx**	**Darbepoetin**	1	66.6	1	66.6	1	55.5
	**Darbepoetin high**	1	83.3	1	66.6	0	33.3
**Kruskal-Wallis**		p<0.05		n.s.		p<0.001	
**Fisher Operation Sham/Unx/Snx**		n.s.		n.s.		n.s.	
**Fisher Medication Control/Darbepoetin**		n.s.		n.s.		n.s.	

*Median and percentage of positive stained endothelia per group.*

#### CD40

**Figure 6:**
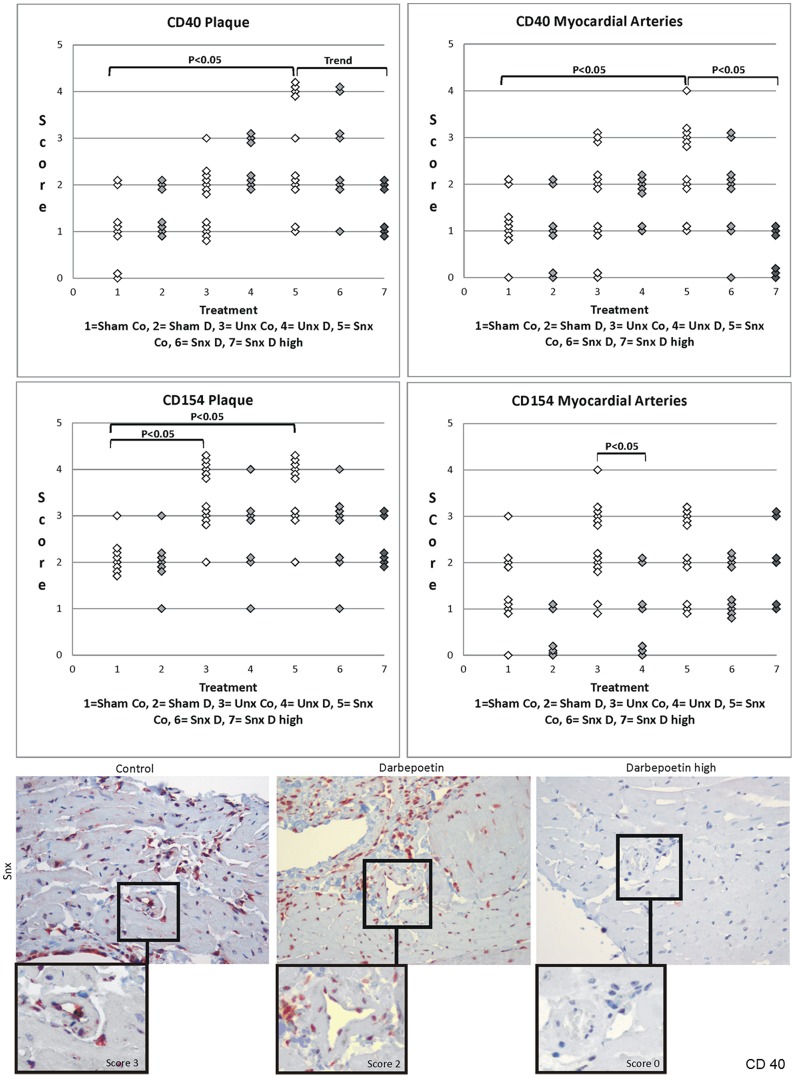
CD 40 and CD 154 plaque and myocardial artery score. Upper rows show CD40 and CD154 staining scores of plaque and intramyocardial arteries. Significant differences are marked with the certain significance level. Lower row shows histological examples of CD40. The Darbepoetin alpha treated Snx animal shows a dose dependently lower CD40 myocardial artery staining score than the control Snx animal.

Protein expression of CD40 in aortic plaques and intramyocardial arteries increased stepwise from Sham to Unx to Snx. The difference between Sham and Snx was significant. Darbepoetin alpha treatment led to lower CD40 expression with the difference being significant in intramyocardial arteries whereas in aortic plaques there was only a tendency for lower values (fig. 6). In the aortic endothelium there was no significant difference (table 9).

#### CD154

In aortic plaques protein expression of CD154 in Unx and Snx animals was significantly higher than in Sham. This effect was a little less pronounced in intramyocardial arteries and therefore not significant. Darbepoetin alpha treatment had no effect on the expression of CD154 in aortic plaques but significantly lowered the protein expression of CD154 in intramyocardial arteries of Unx mice (fig. 6). In the aortic endothelium neither impairment of renal function nor Darbepoetin alpha treatment led to significant changes of CD154 protein expression (table 9).

## Discussion

In the present study possible protective effects of the long acting Epo analogue Darbepoetin alpha on the characteristics of atherosclerosis in apo E knockout mice with chronic renal failure were evaluated. As known by former studies, nephrectomy led to increased expression of markers for oxidative stress and inflammation in the cardiovascular system as well as to more advanced atherosclerotic plaques. Treatment with Darbepoetin alpha significantly lowered plaque stage and in-situ markers of inflammation and oxidative stress. Furthermore hematocrit was elevated dose dependently by the erythropoiesis stimulating agent Darbepoetin. In the high dose group hematocrit values exceeding the upper physiological limit might have negative consequences as well as elevated serum urea.

Most interestingly, intramyocardial arteries and aortic plaques showed a stepwise increase of Nitrotyrosine (NT) protein expression in Unx and Snx. In the aortic endothelium these changes were not seen until Snx. The protein expression of NT and also markers of inflammation correlated with renal function. Other studies with apo E knockout mice also showed higher expression of Nitrotyrosine in aortic plaques in severely reduced renal function [19], [20], [29]. As these studies did not compare different stages of renal function we are the first to show that NT, a marker of oxidative stress, indeed increases proportionally to the degree of renal impairment.

Also patients with CKD show elevated markers of oxidative stress even at mild stages of renal dysfunction [47]. However, the most pronounced effect was seen in patients with severely reduced renal function [47] or patients undergoing dialysis [9].

Thus, our experimental data confirm findings of Cases et al. [18] who showed that oxidative stress starts already in mild renal dysfunction and increases stepwise with renal impairment.

In the present study Darbepoetin alpha treatment exerted its anti-oxidative effect independently from renal function. It significantly lowered in-situ NT expression in the aortic endothelium in Unx and Snx and also in aortic plaques in Snx. This finding in Snx apo E knockout mice, however, is in contrast to findings of Bahlmann et al. in Snx rats where no significant difference in the expression of the anti-oxidative enzyme hemoxygenase under Darbepoetin alpha treatment was seen [42]. This might be due to differences in the animal model or much higher doses of Darbepoetin alpha in our study.

Impaired renal function is a well-known pro-inflammatory state [13]. During an inflammatory reaction several markers are highly expressed in the tissue. One of them is CRP which leads to formation of foam cells [48] and up-regulation of cell adhesion molecules [49]. They contribute to clotting of leucocytes on the endothelium [50]. Also CD40 and CD154 regulate the secretion of cell adhesion molecules [51] and also of MMPs [15], which degrade extracellular matrix and destabilize plaques [52].

In the present study the protein expression of CD154 in aortic plaques and the expression of CRP, ICAM and VCAM were elevated already after Unx. After Snx the expression of CRP, CD40 and VCAM in aortic plaques and of CD40 and CD154 in intramyocardial arteries was elevated, too. Of note, the expression of CRP in intramyocardial arteries correlated with serum urea concentration. Therefore, the higher the degree of impairment of renal function was, the more intense staining for inflammatory markers in vessels and plaques was. When renal function is severely impaired also other markers of inflammation are elevated: Campean et al. showed that the number of CD154-positive cells in coronary plaques of uremic patients was significantly higher than in non-renal control patients. In this tissue the protein expression of CD154 correlated with that of CRP and CD40 [53].

Treatment with Darbepoetin alpha significantly lowered the in-situ expression of CRP in Sham animals, the local expression of CD154 in Unx animals and that of CRP and CD40 in Snx animals in intramyocardial arteries. This indicates an anti-inflammatory effect of Darbepoetin alpha in every stage of renal impairment. Beside this study there is no published work on the anti-inflammatory effect of Darbepoetin alpha in impaired renal function. But Darbepoetin alpha was already shown to lower pro-inflammatory cytokines in patients with chronic cardiac insufficiency or anemia, respectively [54]. In contrast, the anti-inflammatory effect of Epo was widely established in conditions with [55], [56] and without [57], [58] impaired renal function.

In parallel to the decrease in renal function aortic plaque size tended to increase continuously even though not significantly. Of note, aortic plaques of Snx mice showed higher plaque stages with a higher amount of cholesterol crystals. This was not yet seen in Unx mice. Buzello et al. and Bro et al. also observed a steady increase in plaque size from Sham to Unx and Snx and showed that primary in Snx there was a change towards a more inflammatory plaque phenotype with more macrophages and cholesterol deposits [19], [20]. Although we observed a slight increase of plaque size according to renal dysfunction, the differences were not significant. This is a limiting factor of the present study as it is in opposite to many other studies regarding atherosclerosis in Apo E knockout mice [59]–[62]. One reason may be that we used Apo E knockout mice which were reared in our own animal laboratory. So there may be a slightly different gene pool responsible for the missing significance. Other factors could be found in a different timetable of animal surgery and treatment between the studies as well as in a different way of measuring the plaque size. We determined the total plaque volume via microscopy in a distinct section of the ascending aorta as it has been shown that this is the region where Apo E knockout mice mainly develop plaques [63]. Other studies that found significant differences partly evaluated the thoracoabdominal aorta, measured plaque area macroscopically in a lengthwise sectioned aorta or determined plaque area in relation to aortic circumference. This may explain the different results.

In humans it is also likely that even mildly reduced renal function leads to larger plaques, because augmented plaque formation [26] and an increased plaque score [64] were already seen under such conditions. Like in animal models, progression to higher plaque stages was only seen when renal function was markedly impaired, as Schwarz et al. showed in uremic patients [65]. Therefore it is a limitation of the present study that there was no significant difference in plaque size according to renal function.

Treatment with Darbepoetin alpha did not influence plaque size at all. But more dangerous than plaque size, is plaque instability since this can, via rupture of their fibrous cap, lead to thrombembolia and myocardial infarction [14], [66]. The risk of plaque rupture is linked to the total lipid content [66]. In the present study treatment with Darbepoetin alpha significantly lowered plaque stage and presumably the risk of plaque rupture. In Snx animals especially the high dose treatment reduced the content of cholesterol crystals. This effect might have been more evident if the serum cholesterol concentration had also been decreased and the serum lipid concentration had not increased. Sham animals showed no difference. This presumed plaque stabilizing effect of Darbepoetin alpha might also be of clinical importance and seem to be present only when renal function is markedly impaired.

In summary, although the number of animals in some groups was limited, the present study confirmed the negative effects of impaired renal function on the cardiovascular system particularly atherosclerosis most likely via elevated oxidative stress and chronic inflammation. Darbepoetin alpha treatment lowered in-situ markers of oxidative stress and chronic inflammation irrespective of renal function. It also led to lower heart weight and plaque stage and therefore might exert a positive effect on cardiovascular risks in a mouse model of chronic renal failure which may also have implications in patients with CKD. However high dose treatment not only lowered plaque content of cholesterol crystals but also increased heart weight and serum urea, cholesterol and lipid content. Hematocrit was elevated dose dependently, up to unphysiological concentrations in high Darbepoetin alpha dose group. The underlying pathomechanisms, however, which were not addressed in greater detail in the present work, require further studies.

## Supporting Information

Table S1 **Study design.** Detailed table of study design and treatment groups.
(TIF)

Table S2 **Antibodies for immunohistochemistry.** Detailed table of the used antibodies with preparation and incubation guidelines.
(TIF)
